# Use of the PROMIS-10 global health in patients with chronic low back pain in outpatient physical therapy: a retrospective cohort study

**DOI:** 10.1186/s41687-021-00360-8

**Published:** 2021-09-06

**Authors:** Sang S. Pak, Matthew J. Miller, Victor A. Cheuy

**Affiliations:** 1 Department of Physical Therapy and Rehabilitation Science, University of California San Francisco, 1500 Owens St., Suite 400, San Francisco, CA 94158 USA; 2 Division of Geriatrics, University of California, San Francisco, CA USA; 3 Department of Radiology and Biomedical Imaging, University of California, San Francisco, CA USA

**Keywords:** PROMIS, Quality of life, Rehabilitation, Low back pain, Physical therapy

## Abstract

**Background:**

Although evidence-based guidelines for physical therapy for patients with chronic low back pain (cLBP) are available, selecting patient-reported outcome measures to capture complexity of health status and quality of life remains a challenge. PROMIS-10 Global Health (GH) may be used to screen for impactful health risks and enable patient-centered care. The purpose of this study was to investigate the interrelationships between PROMIS-10 GH scores and patient demographics, health status, and healthcare utilization in patients with cLBP who received physical therapy.

**Methods:**

A retrospective review of de-identified electronic health records of patients with cLBP was performed. Data were collected for 328 patients seen from 2017 to 2020 in three physical therapy clinics. Patients were grouped into HIGH and LOW initial assessment scores on the PROMIS-10 Global Physical Health (PH) and Global Mental Health (MH) measures. Outcomes of interest were patient demographics, health status, and healthcare utilization. Mann–Whitney U and chi-square tests were used to determine differences between groups, and binary logistic regression was used to calculate odds ratios (OR) to determine predictors of PH-LOW and MH-LOW group assignments.

**Results:**

The PH-LOW and MH-LOW groups contained larger proportions of patients who were African American, non-Hispanic, and non-commercially insured compared to PH-HIGH and MH-HIGH groups (*p* < .05). The PH-LOW and MH-LOW groups also had a higher Charlson comorbidity index (CCI), higher rates of diabetes and depression, and more appointment cancellations or no-shows (*p* < .05). African American race (OR 2.54), other race (2.01), having Medi-Cal insurance (OR 3.37), and higher CCI scores (OR 1.55) increased the likelihood of being in the PH-LOW group. African American race (OR 3.54), having Medi-Cal insurance (OR 2.19), depression (OR 3.15), kidney disease (OR 2.66), and chronic obstructive pulmonary disease (OR 1.92) all increased the likeihood of being in the MH-LOW group.

**Conclusions:**

Our study identified groups of patients with cLBP who are more likely to have lower PH and MH scores. PROMIS-10 GH provides an opportunity to capture and identify quality of life and global health risks in patients with cLBP. Using PROMIS-10 in physical therapy practice could help identify psychosocial factors and quality of life in the population with cLBP.

## Introduction

Chronic low back pain (cLBP), defined as non-specific back pain lasting at least three months [1], is one of the most common and debilitating health problems in the adult population in the United States [2]. The condition of cLBP is associated with decreased quality of life and represents a tremendous economic and healthcare burden [3–6]. In particular, health-related quality of life (HRQoL)—defined as patient’s self-perceived function and well-being in physical, mental, and social domains of health [7, 8]—is often negatively impacted by cLBP [8]. Previous studies have demonstrated that patients with cLBP are often limited in physical activities [9], have poor mental health status indicated by anxiety and depression [10], and participation restrictions in social activities [11]. HRQoL is one of the core outcome domains recommended for measurement in cLBP population [12]; yet the extent of HRQoL measurement in physical therapy practice has been mixed [13]. Greater adoption of HRQoL measurement within physical therapy practice has potential to improve the care of patients living with cLBP.

The National Institutes of Health (NIH) funded the development of the Patient-Reported Outcome Measurement Information System (PROMIS) for clinicians and researchers to measure health status across multiple domains of HRQoL in disparate health conditions [1, 14–16]. The 10-item PROMIS Global Health survey (PROMIS-10) is measure of health status that spans physical, mental, and social domains from the patient perspective, allowing clinicians and researchers to capture the complex and heterogeneous nature of the populations. The PROMIS-10 (v1.0) has been used as a measure of HRQoL in a variety of chronic health conditions, including back pain, pulmonary disease, and kidney disease [17–19]. The Physical Health (PH) and Mental Health (MH) scores are constructed from the PROMIS-10 [18], and they are standardized to the general population [20]. Worse PH and MH scores are associated with higher rates of hospitalization compared to those patients with higher PH and MH scores in an ambulatory setting [15]. Although recommended for use with people who have low back pain [1, 12, 14, 21], and low back pain accounting for 17.1 million outpatient physical therapy referrals from primary care setting from 1997 to 2010 [22], the use of PROMIS-10 has not specifically been investigated in this specific setting. The high prevalence of people with cLBP who use outpatient physical therapy and the importance of HRQoL suggests that there could be notable physical and mental health risk implications in this clinical setting. Despite these potential implications, studying clinical characteristics and health-related quality of life (HRQoL) in patients with cLBP based on cut scores of PROMIS-10 (PH, MH) in outpatient physical therapy setting could be useful information to improve care. Furthermore, the wide adoption of PROMIS-10 in physical therapy practice could help to identify psychosocial factors and health-related quality of life (HRQoL) in the population with cLBP.

The purpose of this study was to determine group differences in patients with high and low PROMIS-10 PH and MH scores with respect to sociodemographic, health, and health service utilization characteristics in the outpatient physical therapy setting. We then determined the patient characteristics that are independent predictors of low PROMIS-10 PH and MH scores. We hypothesized that patient groups with lower PH and MH scores will have a greater proportion of ethnic and racial minorities, a lower proportion of commercial insurance payer type, higher medical complexity, and higher health service utilization. We further hypothesized that these sociodemographic and health status characteristics will be predictors of lower PROMIS-10 PH and MH scores.

## Methods

### Data source and patients

This retrospective cohort study was conducted using de-identified Electronic Health Record (EHR) data compiled from the clinical data warehouse (CDW) at a large, urban academic medical center (University of California, San Francisco). Data from the CDW were queried for all patients between 18 and 80 years of age with cLBP who had completed PROMIS-10 at initial outpatient physical therapy visit (i.e., physical therapy evaluation) between the dates of January 1, 2017 and June 30, 2020. Low back pain was identified using structured ICD-10 codes for a primary diagnosis of low back pain, dorsalgia, lumbago, radiculopathy, or sciatica. Low back pain was determined to be chronic if the time between original back pain diagnosis and initial physical therapy evaluation was 90 days or greater [1]. If a patient had multiple physical therapy episodes, data from the earliest episode with PROMIS-10 data was used. The last encounter date was defined by the last visit date associated with the original ICD-10 billing diagnosis code used at the initial evaluation visit. Patients with diagnoses of cancer, HIV, or AIDS were excluded to minimize potential confounding effects of these conditions on HRQoL in a population with cLBP [23].

### Outcome measures

Measures were selected based on available evidence for factors that influence HRQoL and outpatient physical therapy utilization in people with cLBP [24–27].

#### Primary measure: PROMIS-10 global health

The PROMIS-10 Global Health survey (v1.0) in electronic or paper format was administered by physical therapists at the initial evaluation visit as part of routine clinical care. The platform for electronic format was through EHR portal, and the paper format was manually transcribed by physical therapists into the EHR system. In both PROMIS-10 Global Health surveys, raw PROMIS-10 scores for Physical Health (PH) and Mental Health (MH) were converted to standardized T-score values [28]. While the PROMIS-10 (v1.0) has been replaced by a more recent version (v1.2), the scoring results between the two versions remain consistent as the questions remain identical to respondents [29]. A T-score of 50 represents the mean of the general population [28], and higher scores indicate better physical and mental health [18, 28]. Evidence suggests “low” PROMIS-10 scores in MH and PH are associated with greater risk for future healthcare utilization [15, 30]. Further, patients with scores categorized as “low” may indicate worse self-rated health that warrants more in depth assessment from physical therapists. Therefore, patients were identified as having poor physical health (PH-LOW) and mental health (MH-LOW) using established T-score cutoffs for fair-to-poor health ratings (PH < 42 and MH < 40) [31]. Patients were identified as having high physical health (PH-HIGH) and mental health (MH-HIGH using established T-score cutoffs for good-to-excellent health ratings (PH^3^42 and MH^3^40). The high and low dichotomized scores were partially based on a previous retrospective cohort study using PROMIS-10 score to identify patients with high risks for future health utilization [15]. However, instead of quartiles to delineate scores [15] our analysis used the T-score thresholds. Further, low scores may indicate poorer self-reported health status that warrants further assessment from physical therapists when evaluating patients with cLBP.

#### Secondary measures: sociodemographics, health status, healthcare utilization

Sociodemographic variables included sex, race (Caucasian, Asian, African American, Other), ethnicity (Hispanic, non-Hispanic), and insurance payer type (commercial, Medi-Cal, Medicare).

Health status was assessed using the Charlson Comorbidity Index (CCI) score and the diagnosis of depression [32]. The CCI measures comorbidity burden by accounting for the number and severity of 19 possible conditions, and is associated with mortality and healthcare utilization [33, 34]. The CCI score was obtained using EHR data to assess the number and severity of comorbid health conditions [32, 33]. Diagnosis of depression is an indicator of mental health, an important part of HRQoL in the cLBP population [35] and was also extracted from electronic health record data.

Healthcare utilization was assessed by metrics of physical therapy and health service utilization. Physical therapy utilization included number of encounters per episode, number of cancellations or no shows, and appointment lead time. Appointment lead time was defined as number of days from the appointment creation date to the appointment date. Health service utilization was assessed using EHR data before the physical therapy evaluation and after the-last physical therapy encounter date. Pre-physical therapy evaluation measures were a history of prescriptions for opioids, and a history of referral for spine imaging within one year prior to the first physical therapy visit date. Post-physical therapy treatment measures were the number of hospital admissions, prescriptions for opioids, and referrals for spine imaging orders within six months after the last encounter date. The last encounter date was assigned when no subsequent appointments were available after 90 days with the same diagnosis. In the event that a patient only had an initial visit, this was also considered the last encounter date.

### Statistical analysis

Median and interquartile range (IQR) were calculated for continuous variables (PH, MH, physical therapy visits, physical therapy cancellations/no-shows, appointment lead time) due to non-normality of the data as determined by the Kolmogorov–Smirnov Test. Sociodemographic, health status, and healthcare utilization characteristics were compared by PH and MH categorization using Mann–Whitney U tests for continuous variables and Chi-square tests for all nominal data, with a significance level of *p* = 0.05. Multivariable logistic regression analyses with backward elimination were performed to determine which sociodemographic and health status variables were associated with PH-LOW and MH-LOW. The initial models included all sociodemographic and health status variables as potential predictor variables and iterative backward selection procedures were then performed until only variables with a *p* value < 0.10 remained in the model. SPSS Statistics V25 (IBM, USA) was used for all analyses. This study protocol was approved by the University of California San Francisco Institutional Review Board.

## Results

**Fig. 1:**
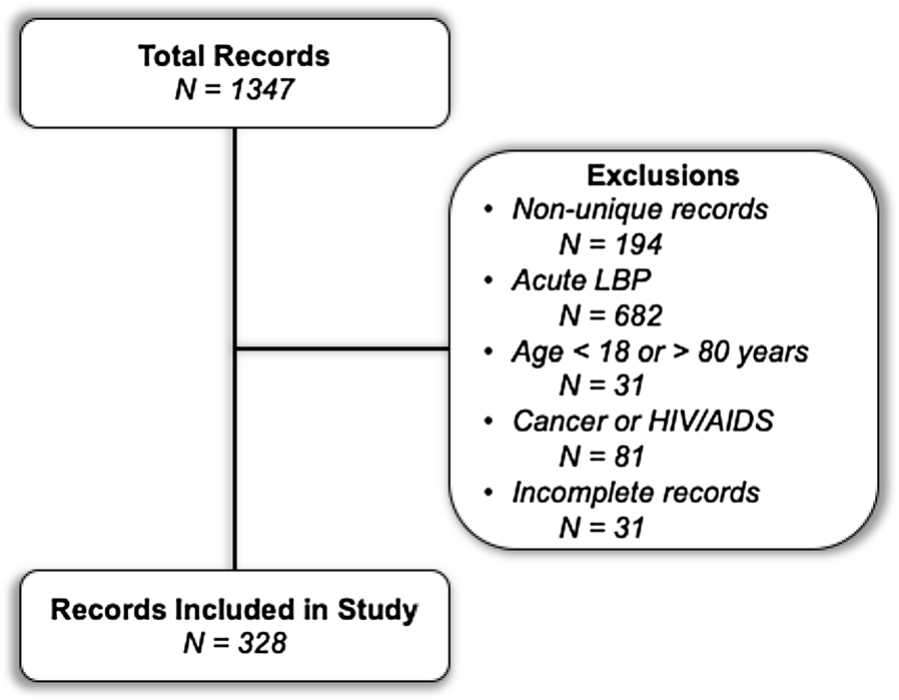
Flow chart of identifying records

The initial data queried from the deidentified clinical data warehouse yielded 1347 patient records. A total of 328 unique patient records remained after excluding non-unique records (N = 194), acute LBP (N = 682), patients younger than 18 years old or over 80 years old (N = 31), Cancer or HIV/AIDS (N = 81) and incomplete records (N = 31) (Fig. 1.). The total cohort included 328 patients with a median age of 52 (Interquartile Range [IQR] 39, 65) and 65% were female, 46% Caucasian, 15% Hispanic, 49% commercial payer type (Table 1). Based on PROMIS-10 PH scores, 179 patients were PH-LOW (median: 34.9 [IQR 29.6–37.4]) and 149 patients were PH-HIGH (median: 47.7 [IQR 44.9–50.8]). Based on PROMIS-10 MH scores, 89 patients were MH-LOW (median: 33.8 [IQR 31.3–36.3]) and 239 patients were MH-HIGH (median: 48.3 [IQR 45.8–53.3]).

**Table 1:** Sociodemographic characteristics*

	Total cohort N = 328	PH-LOW group N = 179	PH-HIGH group N = 149	*p* value^a^	MH-LOW group N = 89	MH-HIGH group N = 239	*p* value^a^
*Sex*				.056			.808
Male	114 (34.8)	54 (30.2)	60 (40.3)		30 (33.7)	84 (35.1)	
Female	214 (65.2)	125 (69.8)	89 (59.7)		59 (66.3)	155 (64.9)	
*Age (years)*^b^	52 (39, 65)	55 (41, 66)	49 (37, 64)	.031	55 (42, 64)	51 (38, 66)	.450
*Race*				.001			< .001
Caucasian	152 (46.3)	73 (40.8)	79 (53.0)		32 (36.0)	120 (50.2)	
Asian	61 (18.6)	27 (15.1)	34 (22.8)		12 (13.5)	49 (20.5)	
African American	50 (15.2)	37 (20.7)	13 (8.7)		27 (30.3)	23 (9.6)	
Other	65 (19.8)	42 (23.5)	23 (15.4)		18 (20.2)	47 (19.7)	
*Ethnicity*				.588			.065
Hispanic	49 (14.9)	25 (14.0)	24 (16.1)		8 (9.0)	41 (17.2)	
Not hispanic	279 (85.1)	154 (86.0)	125 (83.9)		81 (91.0)	198 (82.8)	
*Payer*				< .001			< .001
Commercial	162 (49.4)	67 (37.4)	95 (63.8)		29 (32.6)	133 (55.6)	
Medi-Cal	83 (25.3)	61 (34.1)	22 (14.8)		34 (38.2)	49 (20.5)	
Medicare	83 (25.3)	51 (28.5)	32 (21.5)		26 (29.2)	57 (23.8)	

*Values presented as N (%) unless otherwise stated

^a^Difference between subgroups

^b^Values presented as median (IQR)

Sociodemographic variables are summarized in Table 1. The PH-LOW group was older (55 [41–66] vs 49 [37–64], *p* = 0.031), had a lower proportion of Caucasians (40.8 vs 53.0%, *p* = 0.001), and had a higher proportion of patients with a non-commercial insurance payer type (62.6 vs 36.3%, *p* < 0.001) compared to PH-HIGH (Table 1). The MH-LOW group had a lower proportion of Caucasian patients (36.0 vs 50.2%, *p* < 0.001), and had a higher proportion of patients with a non-commercial insurance payer type (67.4 vs 44.4, *p* < 0.001) compared to MH-HIGH.

The PH-LOW group had a higher CCI score (1 [0, 2] vs 0 [0, 1], *p* < 0.001), a higher prevalence of depression (45.3 vs 28.2%, *p* = 0.001), more physical therapy appointment cancellations or no shows (3 [2, 7] vs 3 [1, 5], *p* = 0.014), shorter appointment lead time (20 [15, 25] vs 22 [17, 28], *p* = 0.031), and a higher incidence of spine imaging ordered post-encounter (17.0 vs 6.7%, *p* = 0.005) compared to PH-HIGH (Table 2). The MH-LOW group had a higher CCI score (1 [0, 3] vs 0 [0, 1], *p* < 0.001), a higher prevalence of depression (59.6 vs 29.3%, *p* < 0.001), less physical therapy visits (3 [1, 6] vs 4 [2, 6], *p* = 0.043), more appointment cancellations or no-shows (4 [2, 9.5] vs 3 [1, 5], *p* = 0.008), and a higher incidence of opioid prescription orders post-encounter (14.0 vs 5.4%, *p* = 0.011) compared to MH-HIGH.

**Table 2:** Health status and utilization*

	Total cohort N = 328	PH-LOW group N = 179	PH-HIGH group N = 149	*p* value^a^	MH-LOW group N = 89	MH-HIGH group N = 239	*p* value^a^
*PROMIS 10 PH*^b^	39.8 (34.9, 44.9)	34.9 (29.6, 37.4)	47.7 (44.9, 50.8)	< .001			
Excellent	5 (1.5)	0 (0.0)	5 (1.5)				
Very good	40 (12.2)	0 (0.0)	40 (12.2)				
Good	104 (31.7)	0 (0.0)	104 (31.7)				
Fair	104 (31.7)	104 (31.7)	0 (0.0)				
Poor	75 (22.9)	75 (22.9)	0 (0.0)				
*PROMIS 10 MH*^b^	45.8 (38.8, 53.3)				33.8 (31.3, 36.3)	48.3 (45.8, 53.3)	< .001
Excellent	35 (10.7)				0 (0.0)	35 (10.7)	
Very Good	127 (38.7)				0 (0.0)	127 (38.7)	
Good	77 (23.5)				0 (0.0)	77 (23.5)	
Fair	57 (17.4)				57 (17.4)	0 (0.0)	
Poor	32 (9.8)				32 (9.8)	0 (0.0)	
*CCI score*^b^	0 (0, 2)	1 (0, 2)	0 (0, 1)	< .001	1 (0, 3)	0 (0, 1)	< .001
0	168 (51.2)	67 (37.4)	101 (67.8)		25 (28.1)	143 (59.8)	
1	76 (23.2)	45 (25.1)	31 (20.8)		27 (30.3)	49 (20.5)	
2	37 (11.3)	27 (15.1)	10 (6.7)		13 (14.6)	24 (10.0)	
3+	47 (14.3)	40 (22.3)	7 (4.7)		24 (27.0)	23 (9.6)	
*Comorbidities*							
Depression	123 (37.5)	81 (45.3)	42 (28.2)	.001	53 (59.6)	70 (29.3)	< .001
Diabetes	57 (17.4)	42 (23.5)	15 (10.1)	.001	27 (30.3)	30 (12.6)	< .001
Kidney disease	43 (13.1)	31 (17.3)	12 (8.1)	.013	23 (25.8)	20 (8.4)	< .001
MI	8 (2.4)	6 (3.4)	2 (1.3)	.240	5 (5.6)	3 (1.3)	.023
CHF	22 (6.7)	19 (10.6)	3 (2.0)	.002	9 (10.1)	13 (5.4)	.132
PVD	29 (8.8)	22 (12.3)	7 (4.7)	.016	9 (10.1)	20 (8.4)	.621
CVD	31 (9.5)	24 (13.4)	7 (4.7)	.007	11 (12.4)	20 (8.4)	.272
Dementia	3 (0.9)	3 (1.7)	0 (0)	.112	2 (2.2)	1 (0.4)	.122
COPD	80 (24.4)	54 (30.2)	26 (17.4)	.008	34 (38.2)	46 (19.2)	< .001
Rheumatic disease	15 (4.6)	11 (6.1)	4 (2.7)	.135	5 (5.6)	10 (4.2)	.580
Peptic ulcer disease	13 (4.0)	10 (5.6)	3 (2.0)	.099	6 (6.7)	7 (2.9)	.116
Liver disease	40 (12.2)	31 (17.3)	9 (6.0)	.002	19 (21.3)	21 (8.8)	.002
Paraplegia	4 (1.2)	4 (2.2)	0 (0.0)	.066	2 (2.2)	2 (0.8)	.301
*Physical therapy utilization*							
Physical therapy visits^b^	3 (2, 6)	3 (1, 6)	3 (2, 6)	.523	3 (1, 6)	4 (2, 6)	.043
Cancellations or no-shows^b^	3 (2, 6)	3 (2, 7)	3 (1, 5)	.014	4 (2, 9.5)	3 (1, 5)	.008
Appointment lead time (days)^b^	21 (16, 27)	20 (15, 25)	22 (17, 28)	.031	19 (15, 25)	21 (16, 27.8)	.065
*Prior utilization within 1 year*							
Opioids ordered	112 (34.1)	69 (38.5)	43 (28.9)	.065	35 (39.3)	7 7(32.2)	.227
Spine imaging ordered	99 (30.2)	49 (27.4)	50 (33.6)	.225	23 (25.8)	76 (31.8)	.296
*Post utilization within 6 months*							
Opioids Ordered	25 (7.7)	18 (10.2)	7 (4.7)	.062	12 (14.0)	13 (5.4)	.011
Spine imaging ordered	40 (12.3)	30 (17.0)	10 (6.7)	.005	14 (16.3)	26 (10.9)	.191
Admittances	9 (2.8)	7 (4.0)	2 (1.3)	.149	4 (4.7)	5 (2.1)	.215

*Values presented as N (%) unless stated otherwise

PH: Physical Health; MH: Mental Health; CCI: Charlson Comorbidity Index; MI, myocardial infarction; CHF, congestive heart failure; PVD, peripheral vascular disease; CVD, cerebrovascular disease; COPD, chronic obstructive pulmonary disease

^a^Difference between subgroups

^b^Values presented as median (IQR)

Results of the logistic regression analyses were expressed as odds ratios (OR [95% CI]). Patient characteristics that were predictive of PH-LOW were African American race (2.54 [1.19–5.41], *p* = 0.016), Other race (2.01 [1.05–3.86], *p* = 0.035), Medi-Cal payer type (3.37 [1.85–6.16], *p* < 0.001), and CCI score (1.55 [1.25–1.93], *p* < 0.001; Table 3). Patient characteristics that were predictive of MH-LOW were African American race (3.54 [1.66–7.58], *p* = 0.001), Medi-Cal payer type (2.19 [1.12–4.27], *p* = 0.022), and diagnoses of depression (3.15 [1.77–5.61], *p* < 0.001), kidney disease (2.66 [1.17–6.08], *p* = 0.020), and COPD (1.92 [1.25–1.93], *p* = 0.039; Table 4).

**Table 3:** Associations of patient characteristics with meeting PH-LOW criteria

Initial model				Final model			
Explanatory variable	OR	95% CI	*p* value	Explanatory variable	OR	95% CI	*p* value
*Sex (reference: Male)*							
Female	1.38	.81–2.35	.231				
Age	1.01	.99–1.03	.264				
*Race (reference: Caucasian)*				*Race (reference: Caucasian)*			
Asian	1.05	.54–2.06	.882	Asian	.98	.52–1.86	.948
African American	2.44	1.10–5.39	.028*	African American	2.54	1.19–5.41	.016*
Other	2.85	1.27–6.38	.011*	Other	2.01	1.05–3.86	.035*
*Ethnicity (reference: Not Hispanic)*							
Hispanic	.57	.24–1.33	.194				
*Payer (reference: Commercial)*				*Payer (reference: Commercial)*			
Medi-Cal	3.10	1.67–5.77	< .001*	Medi-Cal	3.37	1.85–6.16	< .001*
Medicare	.99	.45–2.17	.987	Medicare	1.44	.78–2.67	.250
CCI score	1.18	.78–1.78	.427	CCI score	1.55	1.25–1.93	< .001*
Depression	1.42	.84–2.40	.196				
Diabetes	1.45	.60–3.49	.409				
Kidney disease	.92	.37–2.33	.867				
CHF	1.96	.41–9.35	.397				
PVD	1.19	.38–3.74	.771				
CVD	1.82	.56–5.87	.317				
COPD	1.38	.69–2.77	.362				
Liver disease	1.71	.59–4.92	.324				
Constant	.18		.003*	Constant	.42		< .001*

OR: odds ratio; CI: confidence interval; CCI: Charlson comorbidity index; CHF, congestive heart failure; PVD, peripheral vascular disease; CVD, cerebrovascular disease; COPD, chronic obstructive pulmonary disease

*p < .05

**Table 4:** Associations of patient characteristics with meeting MH-LOW criteria

Initial model				Final model			
Explanatory variable	OR	95% CI	*p* value	Explanatory variable	OR	95% CI	*p* value
*Sex (reference: Male)*							
Female	.72	.39–1.34	.295				
Age	1.00	.97–1.02	.703				
*Race (reference: Caucasian)*				*Race (reference: Caucasian)*			
Asian	1.41	.61–3.27	.420	Asian	1.50	.66–3.42	.337
African American	3.88	1.76–8.53	.001*	African American	3.54	1.66–7.58	.001*
Other	2.18	.87–5.44	.097	Other	2.20	.89–5.45	.087
*Ethnicity (reference: Not Hispanic)*							
Hispanic	.37	.13–1.10	.074	Hispanic	.35	.12–1.04	.059
*Payer (reference: Commercial)*				*Payer (reference: Commercial)*			
Medi-Cal	2.21	1.13–4.33	.021*	Medi-Cal	2.19	1.12–4.27	.022*
Medicare	1.43	.59–3.50	.428	Medicare	1.38	.68–2.78	.371
CCI score	1.01	.76–1.32	.974				
Depression	3.17	1.75–5.75	.000*	Depression	3.15	1.77–5.61	< .001*
Diabetes	1.91	.82–4.47	.134	Diabetes	1.97	.97–4.00	.062
Kidney disease	2.25	.93–5.44	.073	Kidney disease	2.66	1.17–6.08	.020*
MI	1.67	.21–13.13	.629				
COPD	1.92	.98–3.77	.057	COPD	1.92	1.25–1.93	.039*
Liver disease	1.39	.55–3.51	.492				
Constant	.11		.001*	Constant	.07		< .001*

OR: odds ratio; CI: confidence interval; CCI: Charlson comorbidity index; MI: myocardial infarction; COPD, chronic obstructive pulmonary disease

*p < .05

## Discussion

The results of our study show that within a cLBP population, there are significant differences in sociodemographics, health characteristics, and healthcare utilization between patients who score low and high the PROMIS-10 Global Physical Health (PH) and Global Mental Health (MH) domains. In particular, racial background, payer type, and comorbidity status were found to be strong risk factors for low scores across both PROMIS domains.

### Social factors: race and insurance type

Prior studies have shown differences in outcomes contributed by social factors, including race and socioeconomic status (SES), with the disease burden of cLBP [36, 37]. In the present study, there may be an association between patients with lower PH and MH scores and low SES. We observed a higher proportion of African Americans and Medi-Cal insurance payer type to be associated with PH-LOW and MH-LOW scores. Medi-Cal, California’s Medicaid program [38], is a federally and state-funded insurance program covering low-income households and individuals [39]. Previous studies have shown that non-commercial insurance like Medicaid (i.e., Medi-Cal) is a surrogate marker of low SES [40, 41]. Previous studies have also supported associations between lower SES and low HRQoL in a variety of chronic health conditions, such as chronic pain, prostate cancer, hypertension, diabetes mellitus, rheumatism, and heart disease [42–44]. Further research is needed to identify what SES factors contribute to outpatient physical therapy outcomes, including accessibility of physical care site locations and the residential environment.

### Health status

The study findings indicate that a HRQoL is potentially impacted by concomitant disease and there may be a benefit to holistic health approaches. Specifically, higher CCI was a significant risk factor for lower PROMIS-10 physical health scores, highlighting how the cumulative burdens of comorbidities can negatively impact one’s quality of life [45–47]. This finding is consistent with a study by Rothrock et al., in which increased number of chronic conditions in general US population were associated with poorer self-reported HRQoL outcomes measured from PROMIS domains, including physical function [17]. While total comorbidity burden as assessed by the CCI was a significant risk factor for PH-LOW, it was the specific comorbidities of depression, kidney disease, and COPD that were associated with lower PROMIS-10 MH score. Further research is needed to explore the impact of chronic diseases not captured by the CCI that may also influence HRQoL in patients with cLBP in the outpatient physical therapy setting.

### Depression

The association between depression and cLBP is well-documented and it warrants attention in outpatient physical therapy practice [36, 48–51]. Although a meta-analysis by Wong et al. found depression 20% of the population with cLBP [10], we observed depression in 37% of our analytic sample. In the MH-LOW group, nearly 60% of patients had been diagnosed with depression. It is not surprising that a high prevalence of depression was identified in MH-LOW group, given that some global health items in PROMIS-10 are used to assess depressive symptoms [16, 18]. Since depression is an indicator of mental health and an integral part of one’s HRQoL [52], there is an overlap between low PROMIS-10 mental health scores and depression in which they are associated and shown in the MH-LOW group. Therefore, as HRQoL measures like PROMIS-10 are used in physical therapy practice, the group differences in scoring could be helpful to identify and prepare in depth assessments for patients with poor mental health status impacted by depressive symptoms. While risk factors between depression and acute and chronic LBP are not always clear [27, 53], our results underscore the association between depression and cLBP [2, 8, 48–51].

### Health utilization

There are potential cost implications that stem from poor physical and mental health in the cLBP population. Previous studies have shown that CCI holds prognostic value in determining who is likely to incur higher future costs based on the assigned weight of different comorbid health conditions [34, 54]. The economic burdens of no-shows or/and cancellations [55] and the sequelae of procedures like imaging are consistent with the findings of Mugdha et al., namely that increased comorbidities in cLBP are associated with higher health utilization [45]. Furthermore, higher CCI was correlated with higher annual healthcare costs, particularly for patients with Medicare and Medicaid payer sources [34]. The MH-LOW group also had a median of 3 physical therapy visits while MH-HIGH had 4 visits. Although a difference of one visit may not appear to be clinically or administratively relevant, a single visit across the population of people with cLBP could be meaningful for patients who require encounters to address maladaptive beliefs [56] and other psychosocial patterns that persist after therapy. Future work could explore the conceptual framework of PROMIS to help guide clinical decisions and identify potential risks that warrant other appropriate management strategies [56, 57].

## Limitations

Our study is not without limitations. First, we focused only on initial assessment data and we omitted the post-assessment of PROMIS-10 GH scores in our analysis due to incomplete data from our sample at the time of data extraction. Therefore, our ability to understand potential long term changes of HRQoL with outpatient physical therapist interventions were limited. The chronicity of the LBP population was also based on coded data from the de-identified EHR source, which may not always be accurately or coded consistently by the clinical staff. While EHR data are suitable for clinical practice, data are not entered for research purposes, thus diagnosis of depression or other comorbidities may have resulted in intermittent data entry or coding errors. Further, due to small samples in some subgroups, some group differences may not have been detected. A maximum age cutoff of 80 years limits generalizability and the ability to extrapolate these findings to this specific population. Lastly, this was a single site study of patients who received care within a large academic healthcare system, potentially limiting the generalizability to patients who utilize healthcare in a variety of settings. Those who did not directly receive care at site study were not included in the dataset.

## Conclusion

This study found that patients with cLBP who have poor physical and mental health are different across sociodemographic characteristics, comorbidity status, and healthcare utilization, when compared to their patient counterparts who do not score low. In particular, racial background, payer type, and comorbidity status were found to be strong risk factors for low scores across PROMIS-10 physical and mental health domains. The adoption of PROMIS-10 in physical therapy practice could help to identify psychosocial aspects of patients (e.g., depression) and health-related quality of life (HRQoL) in the population with cLBP.

## Data Availability

The dataset generated and/or analysed during the current study are not publicly available due to data owned and compiled by UCSF Information Commons but are available from the corresponding author on reasonable request.

## Abbreviations

HRoQL: Health related quality of life
PROMIS: Patient-reported outcomes measurement information system
CDW: Clinical data warehouse
EHR: Electronic health record
PT: Physical therapy
PH: Physical health
MH: Mental health
PROMIS-10: PROMIS global health
PH-LOW: PROMIS-10 physical health low group
PH-HIGH: PROMIS-10 physical health high group
MH-LOW: PROMIS-10 mental health low group
MH-HIGH: PROMIS-10 mental health high group
